# Supplementary material for: A quantitative comparison of towed-camera and diver-camera transects for monitoring coral reefs
Source: PeerJ. 2021 Apr 14;9:e11090. doi: 10.7717/peerj.11090 (PMC8052974; doi:10.7717/peerj.11090)
Supplement: Supplemental Information 1 [file peerj-09-11090-s001.docx]

# Appendices

###### Additional information on tow-camera surveys

In March 2018 there were

- 3 x 50 m transects at the bommies (C20_BB, C20_BT, M12_BB, M12_BT)
- 3 x 50 m transects on the reef crest (RS1_1-3m, RS2_1-3m, RS3_1-3m)
- 5 x 50 m transects on the reef slope (RS1_1-6m, RS2_1-6m, RS3_1-6m)

In October 2019 there were

- 6 x 20 m transects at the bommies (C20_BB, C20_BT, M12_BB, M12_BT)
- 3 x 50 m transects on the reef crest (RS1_1-3m, RS2_1-3m, RS3_1-3m)
- 5 x 50 m transects on the reef slope (RS1_1-6m, RS2_1-6m, RS3_1-6m)

Figure S 1.1. Photograph of the tow-camera body with key elements indicated from the AIMS Slow Video (SlowVID) standard operating procedure (SOP).


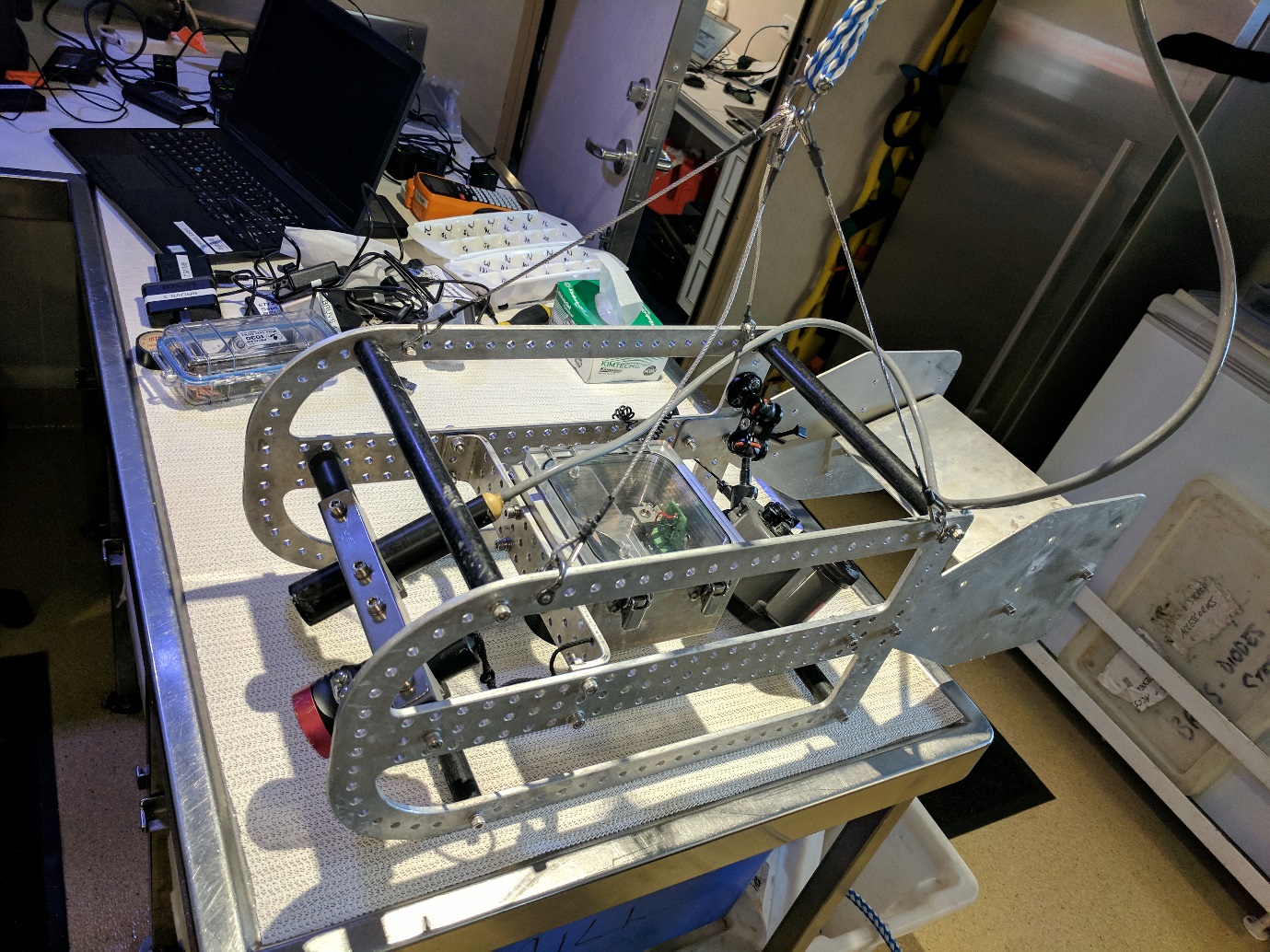


Video Camera

Video Light

Still Camera Housing

Inon Strobe

Cable attachment point


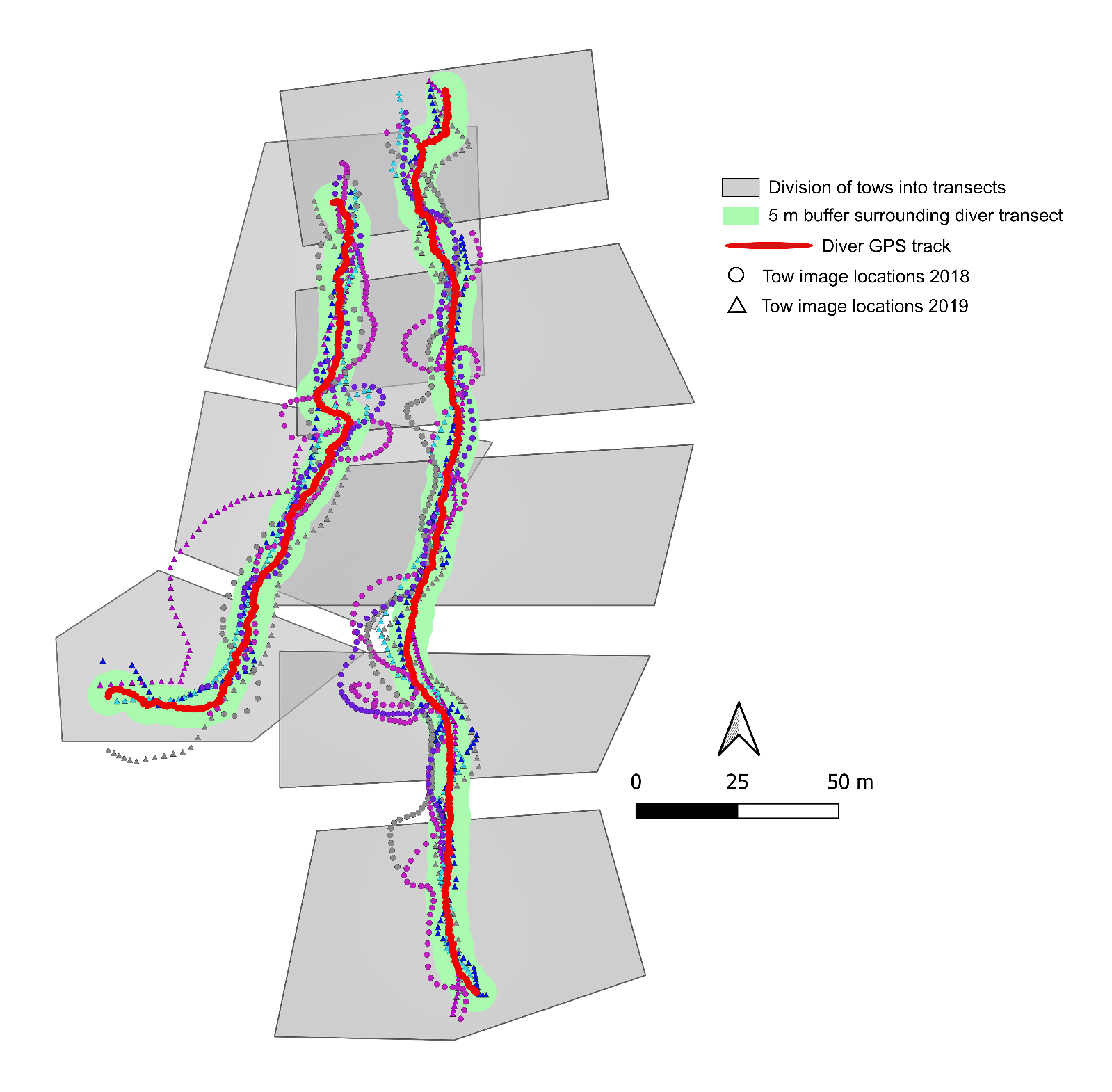


Figure S 1.2. Example of how tows were partitioned into separate transects and image selection. The red line shows the diver GPS track, which the tow followed as close as possible. The circles (2018) and triangles (2019) are the approximate GPS locations of images taken on the tows. Different colours indicate replicate tows. The green shading indicates a 5 m buffer either side of the diver track; only tow-camera images within this buffer were included in the analysis. The grey polygons show how a full site tow was divided into the underlying end-to-end transects.


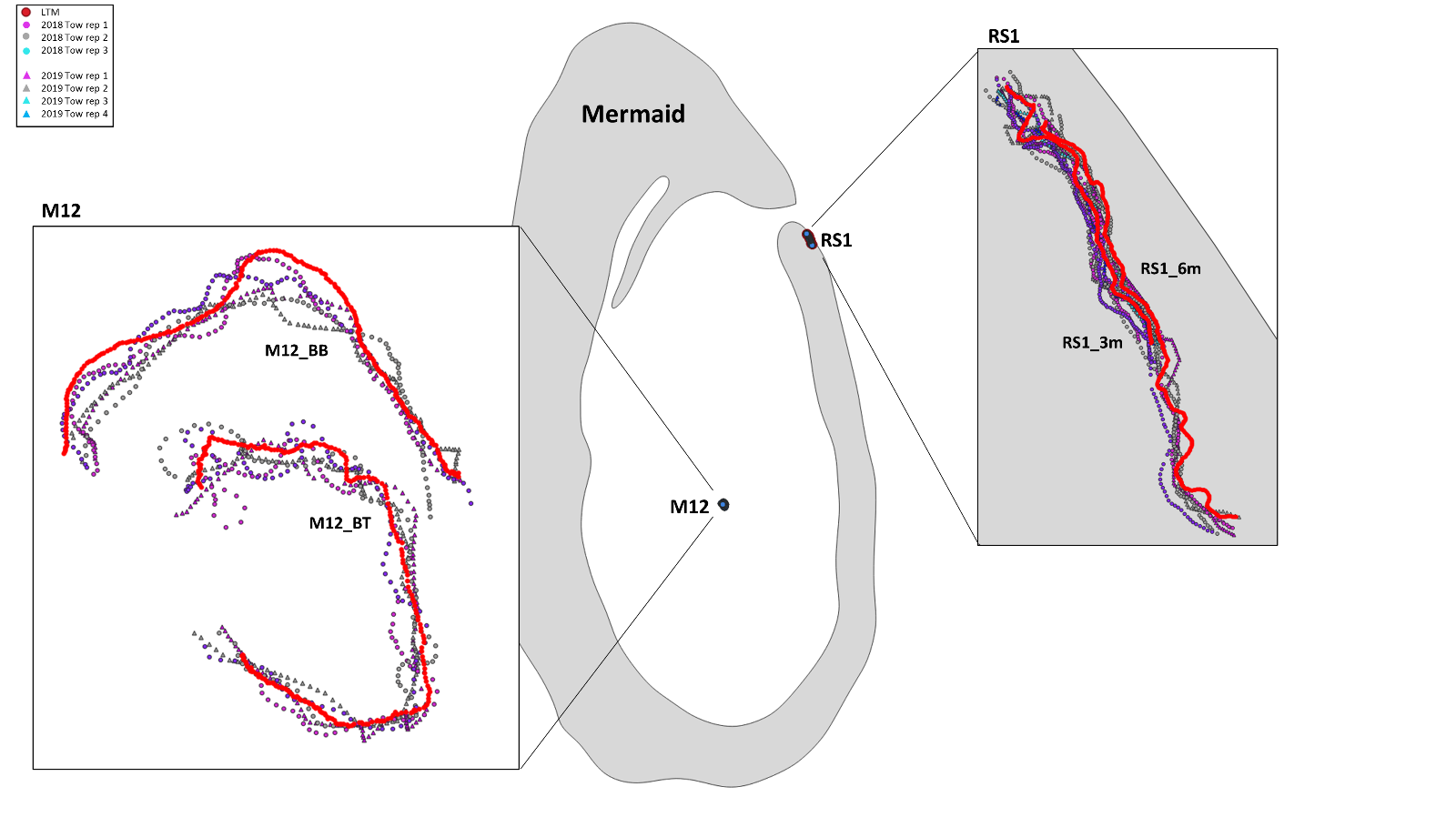

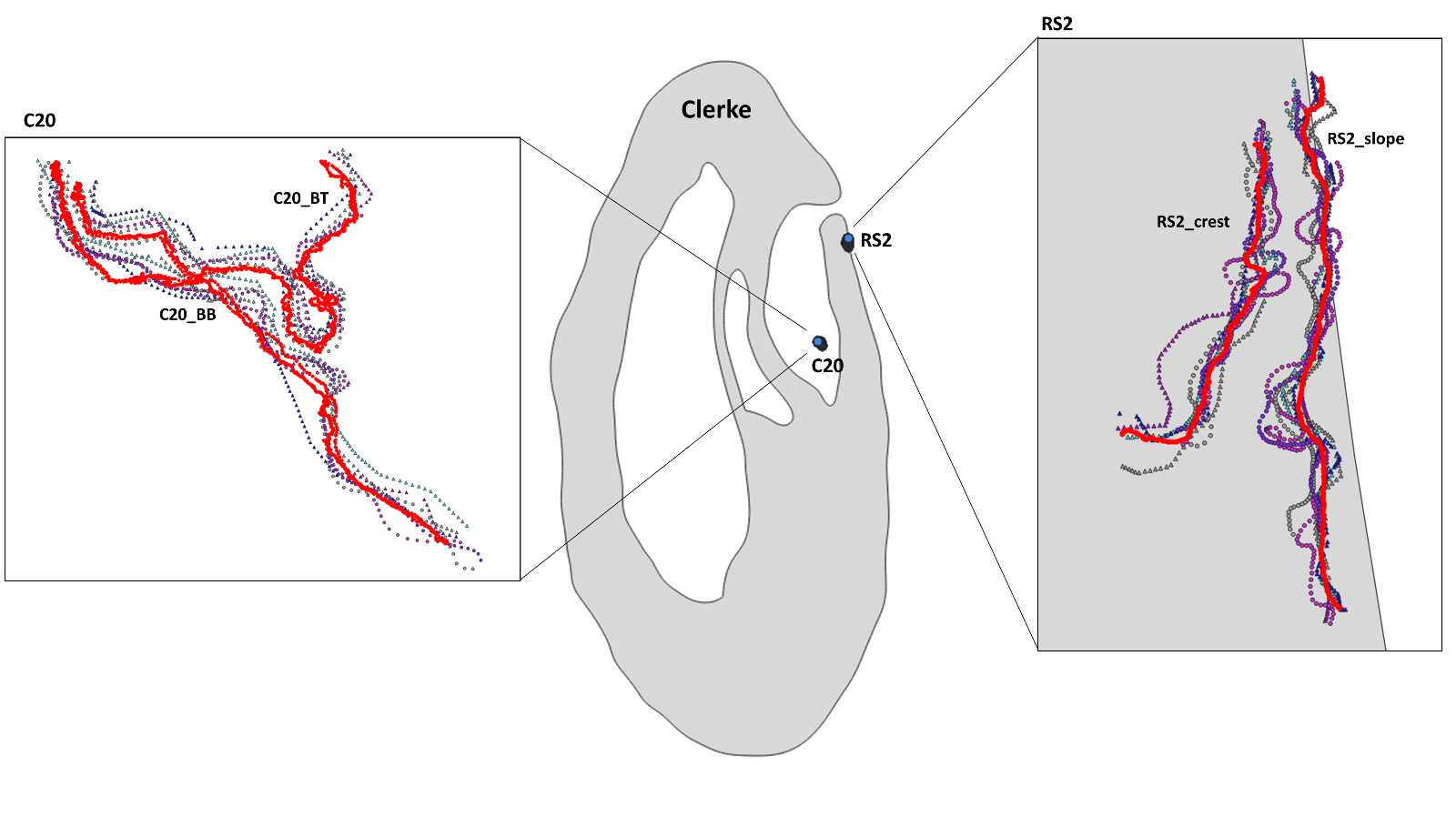


Figure S 1.3. All diver (red) and tow-camera tracks (coloured dots) at the eight Mermaid and Clerke Reef sites.


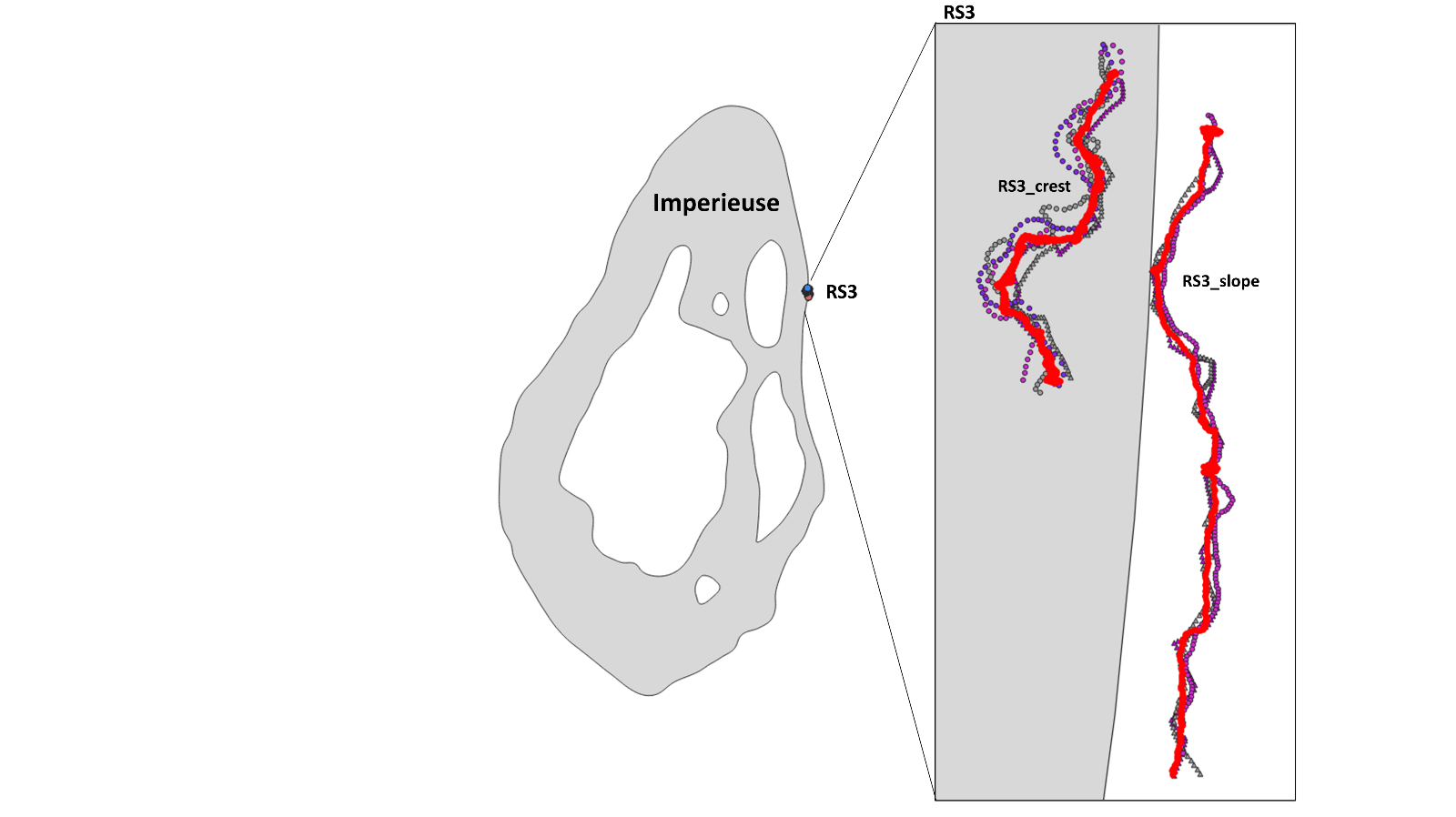


Figure S 1.4. All diver (red) and tow-camera tracks (coloured dots) at the two Imperieuse Reef sites.

Table S 1.1. Summary of images removed due to distance from diver transect and mean distances of the tow-camera images from the fixed-position diver transects before and after image removal.

| Year | Habitat | Site | Total images | Images analysed | Images within buffer | Mean distance all images | Mean distance images within buffer |
| --- | --- | --- | --- | --- | --- | --- | --- |
| 2018 | Lagoon floor | C20_BB | 338 | 241 | 214 | 2.1 | 1.6 |
| 2018 | Bommie | C20_BT | 195 | 142 | 132 | 2.0 | 1.6 |
| 2018 | Bommie | M12_BT | 296 | 207 | 165 | 3.0 | 2.0 |
| 2018 | Lagoon floor | M12_BB | 277 | 200 | 134 | 3.8 | 2.2 |
| 2018 | Reef crest | RS1_1_3m | 361 | 260 | 144 | 4.7 | 2.6 |
| 2018 | Reef slope | RS1_1_6m | 390 | 280 | 163 | 5.6 | 2.4 |
| 2018 | Reef crest | RS2_1_3m | 353 | 250 | 179 | 3.4 | 1.9 |
| 2018 | Reef slope | RS2_1_6m | 454 | 320 | 194 | 5.2 | 2.5 |
| 2018 | Reef crest | RS3_1_3m | 243 | 173 | 121 | 3.5 | 2.0 |
| 2018 | Reef slope | RS3_1_6m | 214 | 153 | 138 | 2.6 | 2.1 |
| 2019 | Lagoon floor | C20_BB | 490 | 490 | 389 | 2.9 | 1.8 |
| 2019 | Bommie | C20_BT | 428 | 428 | 335 | 3.5 | 2.0 |
| 2019 | Bommie | M12_BB | 273 | 273 | 156 | 5.5 | 1.9 |
| 2019 | Lagoon floor | M12_BT | 265 | 265 | 234 | 3.1 | 1.9 |
| 2019 | Reef crest | RS1_1_3m | 588 | 588 | 476 | 2.9 | 1.9 |
| 2019 | Reef slope | RS1_1_6m | 401 | 401 | 312 | 4.2 | 2.0 |
| 2019 | Reef crest | RS2_1_3m | 502 | 502 | 259 | 11.0 | 2.1 |
| 2019 | Reef slope | RS2_1_6m | 660 | 660 | 583 | 2.3 | 1.6 |
| 2019 | Reef crest | RS3_1_3m | 224 | 224 | 198 | 2.5 | 2.0 |
| 2019 | Reef slope | RS3_1_6m | 363 | 363 | 328 | 2.0 | 1.5 |

###### Additional information on covariates

Table S 2.1. Mean, standard deviation (SD) and range of slope and depths of each site

| **Site** | **Mean slope** | **SD slope** | **Maximum slope** | **Minimum slope** | **Mean depth** | **SD depth** | **Maximum depth** | **Minimum depth** |
| --- | --- | --- | --- | --- | --- | --- | --- | --- |
| **C20_BB** | **15.62922** | **6.973706** | **36.05087** | **3.144965** | **-3.62301** | **1.351265** | **-0.62757** | **-6.69246** |
| **C20_BT** | **14.07977** | **9.566393** | **33.74439** | **0.63958** | **-1.17078** | **0.961841** | **-0.18745** | **-5.91893** |
| **M12_BB** | **25.43189** | **4.540996** | **31.87066** | **16.13121** | **-9.82217** | **0.974678** | **-7.82711** | **-12.2902** |
| **M12_BT** | **7.659009** | **3.947725** | **17.73629** | **1.506977** | **-3.43983** | **0.602143** | **-2.43824** | **-5.02123** |
| **RS1_1_3m** | **27.23049** | **12.8781** | **45.94009** | **0.822844** | **-4.9376** | **1.733825** | **-2.27842** | **-8.40957** |
| **RS1_1_6m** | **38.76374** | **11.11776** | **58.77383** | **10.35546** | **-8.59698** | **2.426796** | **-3.99982** | **-13.5148** |
| **RS2_1_3m** | **2.857542** | **1.609829** | **8.867118** | **0.8115** | **-3.40515** | **0.702373** | **-1.82304** | **-4.33967** |
| **RS2_1_6m** | **17.0146** | **5.259219** | **30.06892** | **6.809129** | **-8.31598** | **1.86423** | **-5.45439** | **-13.6511** |
| **RS3_1_3m** | **7.439029** | **2.468455** | **13.65593** | **2.991309** | **-4.29808** | **0.703253** | **-2.59782** | **-5.68995** |
| **RS3_1_6m** | **3.960932** | **2.812719** | **11.11246** | **0.452653** | **-7.22252** | **0.600925** | **-5.88012** | **-8.31287** |

###### Additional statistics

Table S 3.1. Pairwise comparison for the term method x habitat for pairs of the levels of factor method, i.e. tow-camera versus diver-camera, within each habitat level.

| Benthic community | | | |
| --- | --- | --- | --- |
| Habitat | t (perm) | P (perm) | Permutations |
| Crest | 1.7072 | 0.0375 | 9961 |
| Lagoon bommie | 3.758 | 0.0001 | 9962 |
| Lagoon floor | 4.999 | 0.0001 | 9972 |
| Slope | 2.4707 | 0.0001 | 9957 |
| Coral community | | | |
| Crest | 1.9458 | 0.0006 | 9941 |
| Lagoon bommie | 1.1772 | 0.247 | 9958 |
| Lagoon floor | 1.1046 | 0.3199 | 9947 |
| Slope | 2.3332 | 0.0001 | 9943 |

Table S 3.2. Mean and standard error of cover across all transects for each benthic and coral group.

|  | Diver mean | Diver SE | Tow mean | Tow SE |
| --- | --- | --- | --- | --- |
| **Benthic groups** |  |  |  |  |
| Abiotic | 8.33 | 1.27 | 16.15 | 2.28 |
| Crustose coralline algae | 51.30 | 1.61 | 36.69 | 1.52 |
| Hard coral | 37.56 | 1.79 | 32.56 | 1.82 |
| Unidentified substratum | 1.29 | 0.22 | 12.58 | 1.56 |
| Macroalgae | 3.58 | 0.42 | 2.53 | 0.27 |
| Other organisms | 0.87 | 0.10 | 0.85 | 0.12 |
| Soft coral | 2.28 | 0.38 | 2.23 | 0.43 |
| Sponge | 1.60 | 0.22 | 1.17 | 0.18 |
| **Coral groups** |  |  |  |  |
| Branching *Acropora* | 6.31 | 0.82 | 6.32 | 0.90 |
| Digitate and corymbose *Acropora* | 7.02 | 0.58 | 4.88 | 0.40 |
| Tabular *Acropora* | 3.22 | 0.39 | 4.28 | 0.41 |
| *Astreopora* | 0.93 | 0.16 | 0.84 | 0.12 |
| Unidentified coral | 0.37 | 0.00 | 0.71 | 0.10 |
| *Diploastrea* | 3.41 | 0.81 | 1.71 | 0.39 |
| Encrusting corals | 10.26 | 0.69 | 8.54 | 0.63 |
| Foliose corals | 1.11 | 0.31 | 0.91 | 0.23 |
| Fungiidae | 1.62 | 0.24 | 1.33 | 0.22 |
| *Isopora* | 5.05 | 0.50 | 4.59 | 0.39 |
| Merulinidae | 4.18 | 0.30 | 3.03 | 0.26 |
| *Millepora* | 3.37 | 0.38 | 2.15 | 0.20 |
| *Pavona* | 0.65 | 0.15 | 0.50 | 0.08 |
| Pocilloporidae | 3.50 | 0.28 | 2.92 | 0.26 |
| *Porites* | 2.01 | 0.23 | 2.01 | 0.23 |
| Uncommon | 0.99 | 0.14 | 0.95 | 0.09 |


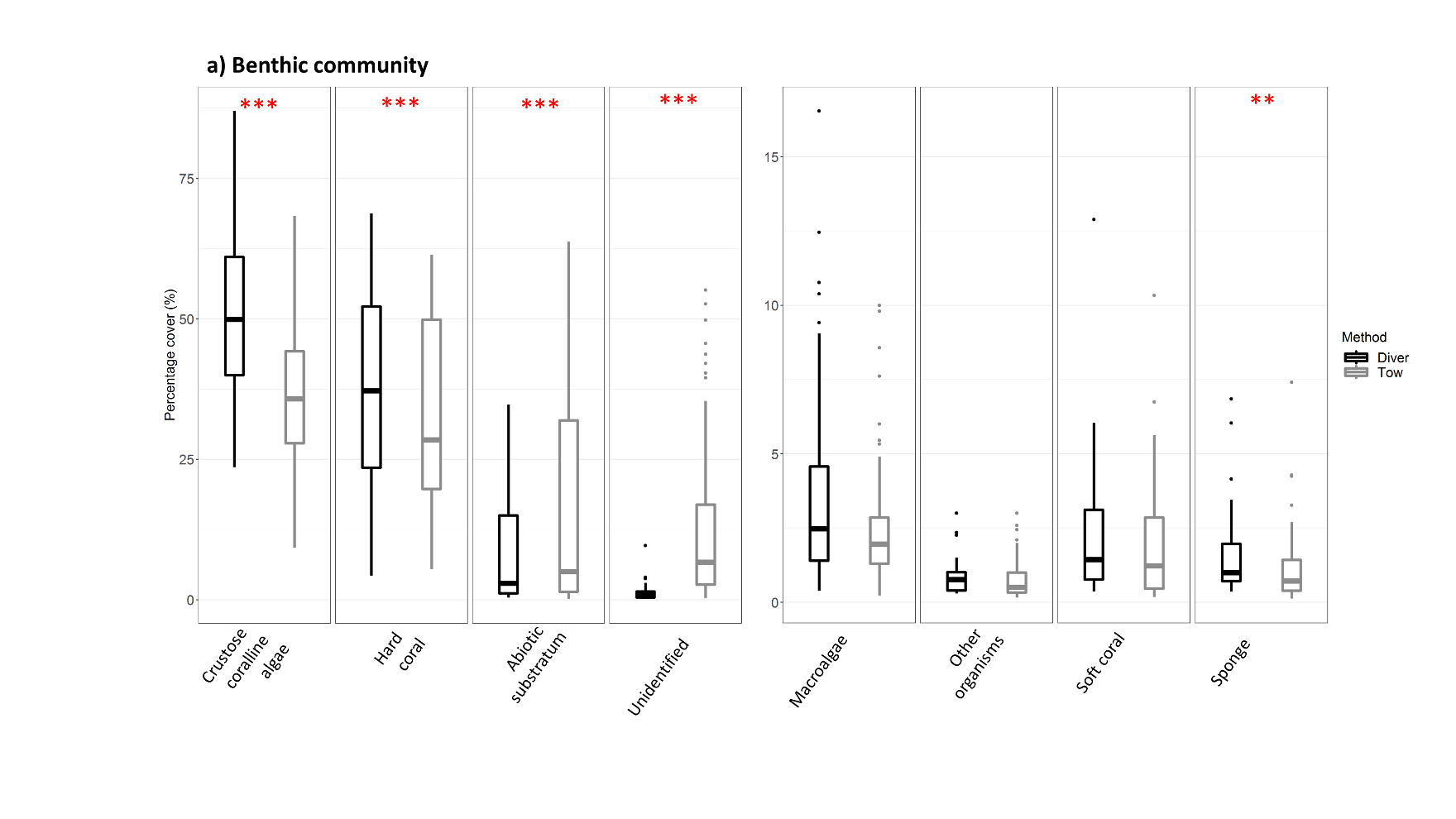


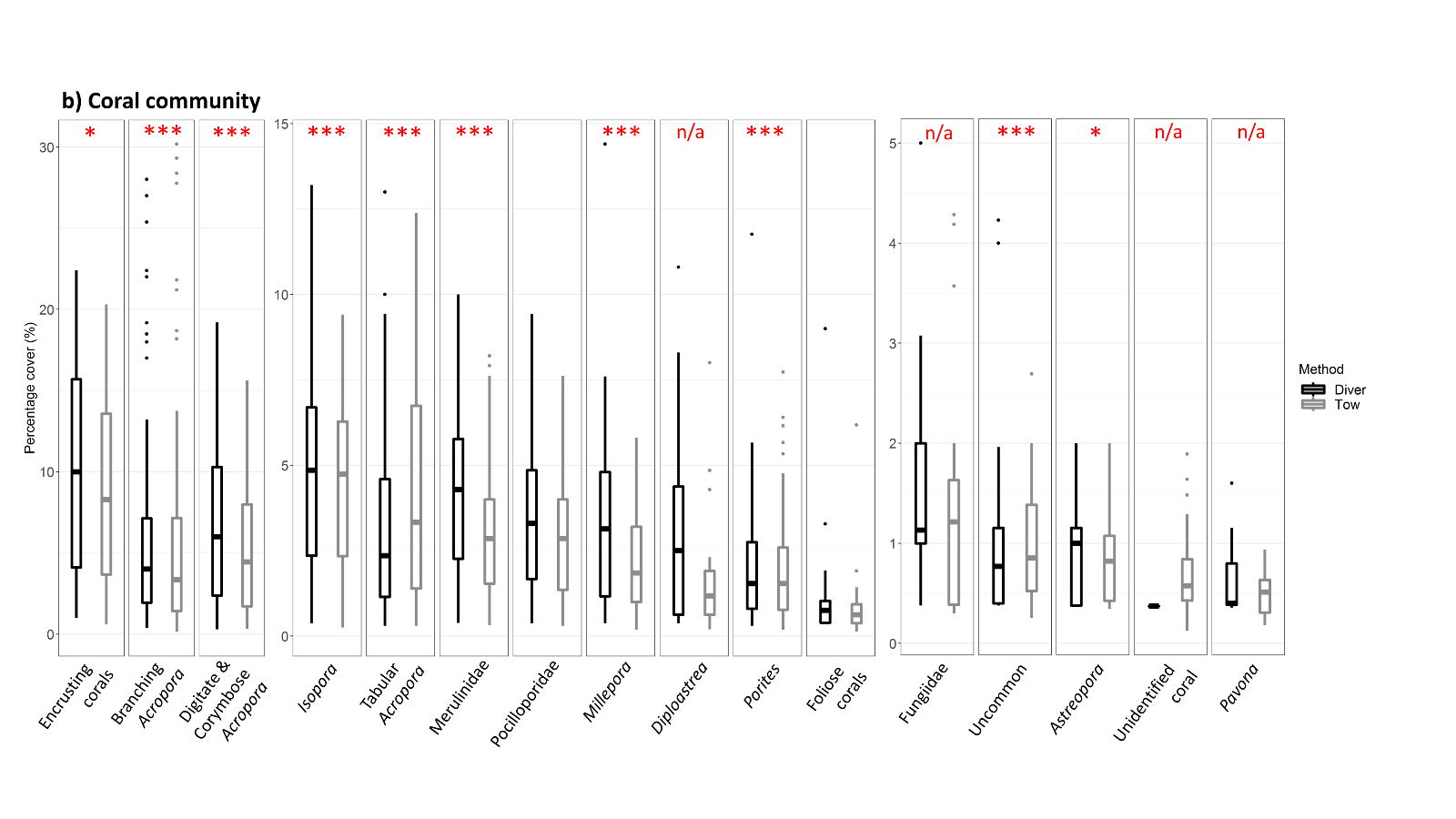


Figure S 3.1. Boxplots of each of the a) benthic and b) coral community categories for the two survey methods averaged across all transects (n = 168 transects spread over 10 sites and two years of surveys). Red asterisks indicate a significant difference between the two methods (***: p < 0.001, **p < 0.01, * p < 0.05) as determined from binomial generalised linear mixed-effects models.

Table S 3.3. Intercepts and test statistics from linear models for each benthic and coral group. The intercept gives the prediction of Δcover (diver -tow-camera) when all other variables in the models (see Table 3.3) were mean centred. Blue text (positive) indicates the diver detected more cover of a group, while red text indicates the tow-camera detected a higher cover.

| **Benthic groups** |  |  |  |  |
| --- | --- | --- | --- | --- |
| Group | Estimate | Std. error | t-value | P-value |
| Crustose coralline algae | 13.609 | 0.432 | 31.537 | <0.001 |
| Hard coral | 4.246 | 0.359 | 11.828 | <0.001 |
| Abiotic | -10.274 | 0.429 | -23.945 | <0.001 |
| Unidentified substratum | -7.771 | 0.426 | -18.234 | <0.001 |
| Macroalgae | 0.159 | 0.117 | 1.354 | 0.176 |
| Other organisms | 0.067 | 0.037 | 1.804 | 0.072 |
| Soft coral | 0.157 | 0.078 | 2.026 | 0.043 |
| Sponge | -0.195 | 0.08 | -2.447 | 0.015 |
| **Coral taxa** |  |  |  |  |
| Group | Estimate | Std. error | t-value | P-value |
| Branching *Acropora* | -0.456 | 0.157 | -2.902 | 0.004 |
| Digitate and corymbose *Acropora* | 1.996 | 0.134 | 14.86 | <0.001 |
| Tabular *Acropora* | -1.215 | 0.121 | -10.051 | <0.001 |
| *Astreopora* | -0.035 | 0.018 | -2.019 | 0.044 |
| Encrusting corals | 0.578 | 0.175 | 3.3 | 0.001 |
| Foliose corals | -0.067 | 0.046 | -1.462 | 0.144 |
| *Isopora* | 0.582 | 0.091 | 6.371 | <0.001 |
| Merulinidae | 1.071 | 0.091 | 11.73 | <0.001 |
| *Millepora* | 0.489 | 0.093 | 5.27 | <0.001 |
| Pocilloporidae | 0.24 | 0.07 | 3.443 | <0.001 |
| *Porites* | 0.427 | 0.064 | 6.683 | <0.001 |
| Uncommon | 0.156 | 0.038 | 4.104 | <0.001 |

Table S 3.4. Test statistics from linear models for each benthic and coral group. The first term is the intercept for the crest habitat, at Clerke reef, in 2018, and all other estimates reference to this estimate. Colours match to Figure 3 in the main manuscript. Blue text indicates a positive estimate while red text is a negative estimate.

| **Benthic groups** |  |  |  |  |  |
| --- | --- | --- | --- | --- | --- |
| group | Term | Estimate | Std. error | t-value | P-value |
| Crustose coralline algae | Slope_Clerke_2018 | -26.092 | 1.936 | -13.477 | <0.001 |
| Crustose coralline algae | log (images) | -3.764 | 0.83 | -4.535 | <0.001 |
| Crustose coralline algae | depth | 2.373 | 0.256 | 9.271 | <0.001 |
| Crustose coralline algae | Δ Crest | 7.771 | 1.352 | 5.748 | <0.001 |
| Crustose coralline algae | Δ Bommie | 16.827 | 1.824 | 9.226 | <0.001 |
| Crustose coralline algae | Δ Lagoon floor | 23.852 | 1.072 | 22.26 | <0.001 |
| Crustose coralline algae | Δ Imperieuse | -2.222 | 1.105 | -2.012 | 0.045 |
| Crustose coralline algae | Δ Mermaid | 11.544 | 1.039 | 11.109 | <0.001 |
| Crustose coralline algae | Δ 2019 | 22.533 | 0.946 | 23.815 | <0.001 |
| Hard coral | Slope_Clerke_2018 | 9.282 | 1.935 | 4.796 | <0.001 |
| Hard coral | log (slope) | 3.122 | 0.484 | 6.448 | <0.001 |
| Hard coral | log (images) | -2.822 | 0.718 | -3.93 | <0.001 |
| Hard coral | depth | -2.072 | 0.216 | -9.583 | <0.001 |
| Hard coral | Δ Crest | -6.311 | 1.133 | -5.568 | <0.001 |
| Hard coral | Δ Bommie | -10.037 | 1.533 | -6.546 | <0.001 |
| Hard coral | Δ Lagoon floor | -10.516 | 0.891 | -11.798 | <0.001 |
| Hard coral | Δ Imperieuse | 5.668 | 1.01 | 5.612 | <0.001 |
| Hard coral | Δ Mermaid | 8.137 | 0.917 | 8.873 | <0.001 |
| Hard coral | Δ 2019 | 2.945 | 0.799 | 3.685 | <0.001 |
| Abiotic | Slope_Clerke_2018 | 20.162 | 2.313 | 8.716 | <0.001 |
| Abiotic | log (slope) | 2.51 | 0.579 | 4.336 | <0.001 |
| Abiotic | log (images) | 6.137 | 0.858 | 7.15 | <0.001 |
| Abiotic | depth | -2.112 | 0.258 | -8.174 | <0.001 |
| Abiotic | Δ Crest | -11.183 | 1.355 | -8.255 | <0.001 |
| Abiotic | Δ Bommie | -24.01 | 1.833 | -13.101 | <0.001 |
| Abiotic | Δ Lagoon floor | -27.298 | 1.065 | -25.622 | <0.001 |
| Abiotic | Δ Imperieuse | -3.18 | 1.207 | -2.634 | 0.009 |
| Abiotic | Δ Mermaid | -7.024 | 1.096 | -6.408 | <0.001 |
| Abiotic | Δ 2019 | -11.802 | 0.955 | -12.357 | <0.001 |
| Unidentified substratum | Slope_Clerke_2018 | -8.6 | 2.298 | -3.743 | <0.001 |
| Unidentified substratum | log (slope) | -6.206 | 0.575 | -10.793 | <0.001 |
| Unidentified substratum | log (images) | 2.326 | 0.853 | 2.728 | 0.007 |
| Unidentified substratum | depth | 1.957 | 0.257 | 7.624 | <0.001 |
| Unidentified substratum | Δ Crest | 12.003 | 1.346 | 8.919 | <0.001 |
| Unidentified substratum | Δ Bommie | 23.761 | 1.82 | 13.052 | <0.001 |
| Unidentified substratum | Δ Lagoon floor | 14.473 | 1.058 | 13.676 | <0.001 |
| Unidentified substratum | Δ Imperieuse | 3.715 | 1.199 | 3.098 | 0.002 |
| Unidentified substratum | Δ Mermaid | -13.026 | 1.089 | -11.964 | <0.001 |
| Unidentified substratum | Δ 2019 | -9.562 | 0.949 | -10.079 | <0.001 |
| Macroalgae | Slope_Clerke_2018 | 2.946 | 0.633 | 4.655 | <0.001 |
| Macroalgae | log (slope) | 0.289 | 0.158 | 1.822 | 0.069 |
| Macroalgae | log (images) | -0.788 | 0.235 | -3.355 | <0.001 |
| Macroalgae | depth | 0.127 | 0.071 | 1.793 | 0.073 |
| Macroalgae | Δ Crest | -1.289 | 0.371 | -3.477 | <0.001 |
| Macroalgae | Δ Bommie | -3.743 | 0.501 | -7.465 | <0.001 |
| Macroalgae | Δ Lagoon floor | -0.125 | 0.292 | -0.429 | 0.668 |
| Macroalgae | Δ Imperieuse | -3.159 | 0.33 | -9.562 | <0.001 |
| Macroalgae | Δ Mermaid | 0.69 | 0.3 | 2.3 | 0.022 |
| Macroalgae | Δ 2019 | -4.105 | 0.261 | -15.707 | <0.001 |
| Other organisms | Slope_Clerke_2018 | -0.81 | 0.16 | -5.065 | <0.001 |
| Other organisms | log (slope) | 0.336 | 0.051 | 6.592 | <0.001 |
| Other organisms | log (images) | -0.618 | 0.076 | -8.165 | <0.001 |
| Other organisms | Δ Crest | 0.161 | 0.091 | 1.783 | 0.075 |
| Other organisms | Δ Bommie | -0.299 | 0.103 | -2.911 | 0.004 |
| Other organisms | Δ Lagoon floor | 0.526 | 0.09 | 5.847 | <0.001 |
| Other organisms | Δ Imperieuse | -0.015 | 0.106 | -0.14 | 0.889 |
| Other organisms | Δ Mermaid | -0.495 | 0.083 | -5.958 | <0.001 |
| Other organisms | Δ 2019 | 0.336 | 0.085 | 3.962 | <0.001 |
| Soft coral | Slope_Clerke_2018 | 0.539 | 0.348 | 1.549 | 0.122 |
| Soft coral | log (images) | -0.306 | 0.149 | -2.053 | 0.04 |
| Soft coral | depth | -0.087 | 0.046 | -1.888 | 0.059 |
| Soft coral | Δ Crest | 0.121 | 0.243 | 0.498 | 0.619 |
| Soft coral | Δ Bommie | 0.094 | 0.328 | 0.286 | 0.775 |
| Soft coral | Δ Lagoon floor | 0.657 | 0.193 | 3.412 | <0.001 |
| Soft coral | Δ Imperieuse | 0.2 | 0.199 | 1.009 | 0.313 |
| Soft coral | Δ Mermaid | -0.406 | 0.187 | -2.171 | 0.03 |
| Soft coral | Δ 2019 | -0.071 | 0.17 | -0.416 | 0.678 |
| Sponge | Slope_Clerke_2018 | 1.646 | 0.429 | 3.839 | <0.001 |
| Sponge | log (slope) | 0.38 | 0.107 | 3.543 | <0.001 |
| Sponge | log (images) | -0.342 | 0.159 | -2.152 | 0.032 |
| Sponge | depth | -0.224 | 0.048 | -4.672 | <0.001 |
| Sponge | Δ Crest | -1.164 | 0.251 | -4.635 | <0.001 |
| Sponge | Δ Bommie | -2.42 | 0.34 | -7.124 | <0.001 |
| Sponge | Δ Lagoon floor | -1.571 | 0.197 | -7.953 | <0.001 |
| Sponge | Δ Imperieuse | -0.631 | 0.224 | -2.821 | 0.005 |
| Sponge | Δ Mermaid | 0.318 | 0.203 | 1.564 | 0.118 |
| Sponge | Δ 2019 | -0.15 | 0.177 | -0.849 | 0.396 |
| **Coral taxa** |  |  |  |  |  |
| group | Term | Estimate | Std. error | t-value | P-value |
| Branching *Acropora* | Slope_Clerke_2018 | 1.547 | 0.856 | 1.808 | 0.071 |
| Branching *Acropora* | log (slope) | -0.747 | 0.192 | -3.893 | <0.001 |
| Branching *Acropora* | depth | -0.198 | 0.098 | -2.019 | 0.044 |
| Branching *Acropora* | Δ Crest | -0.545 | 0.48 | -1.137 | 0.256 |
| Branching *Acropora* | Δ Bommie | 1.226 | 0.705 | 1.739 | 0.082 |
| Branching *Acropora* | Δ Lagoon floor | -3.205 | 0.412 | -7.772 | <0.001 |
| Branching *Acropora* | Δ Imperieuse | 1.072 | 0.382 | 2.805 | 0.005 |
| Branching *Acropora* | Δ Mermaid | 2.779 | 0.374 | 7.422 | <0.001 |
| Branching *Acropora* | Δ 2019 | 0.639 | 0.242 | 2.635 | 0.009 |
| Digitate and corymbose *Acropora* | Slope_Clerke_2018 | 1.925 | 0.731 | 2.632 | 0.009 |
| Digitate and corymbose *Acropora* | log (slope) | -0.582 | 0.164 | -3.551 | <0.001 |
| Digitate and corymbose *Acropora* | depth | 0.351 | 0.084 | 4.173 | <0.001 |
| Digitate and corymbose *Acropora* | Δ Crest | 0.99 | 0.41 | 2.414 | 0.016 |
| Digitate and corymbose *Acropora* | Δ Bommie | -0.401 | 0.602 | -0.666 | 0.506 |
| Digitate and corymbose *Acropora* | Δ Lagoon floor | -1.663 | 0.353 | -4.718 | <0.001 |
| Digitate and corymbose *Acropora* | Δ Imperieuse | -2.027 | 0.327 | -6.208 | <0.001 |
| Digitate and corymbose *Acropora* | Δ Mermaid | -2.878 | 0.32 | -8.992 | <0.001 |
| Digitate and corymbose *Acropora* | Δ 2019 | 2.581 | 0.207 | 12.455 | <0.001 |
| Tabular *Acropora* | Slope_Clerke_2018 | -1.223 | 0.669 | -1.828 | 0.068 |
| Tabular *Acropora* | log (slope) | 0.348 | 0.152 | 2.29 | 0.022 |
| Tabular *Acropora* | log (images) | -0.738 | 0.255 | -2.897 | 0.004 |
| Tabular *Acropora* | depth | -0.471 | 0.076 | -6.199 | <0.001 |
| Tabular *Acropora* | Δ Crest | 0.076 | 0.373 | 0.203 | 0.839 |
| Tabular *Acropora* | Δ Bommie | 1.446 | 0.544 | 2.658 | 0.008 |
| Tabular *Acropora* | Δ Lagoon floor | 2.245 | 0.321 | 6.993 | <0.001 |
| Tabular *Acropora* | Δ Imperieuse | 1.419 | 0.301 | 4.714 | <0.001 |
| Tabular *Acropora* | Δ Mermaid | 2.87 | 0.294 | 9.762 | <0.001 |
| Tabular *Acropora* | Δ 2019 | -0.7 | 0.275 | -2.543 | 0.011 |
| *Astreopora* | Slope_Clerke_2018 | 0.258 | 0.096 | 2.694 | 0.007 |
| *Astreopora* | log (slope) | 0.047 | 0.021 | 2.183 | 0.029 |
| *Astreopora* | depth | -0.049 | 0.011 | -4.439 | <0.001 |
| *Astreopora* | Δ Crest | -0.147 | 0.054 | -2.741 | 0.006 |
| *Astreopora* | Δ Bommie | -0.118 | 0.079 | -1.502 | 0.133 |
| *Astreopora* | Δ Lagoon floor | -0.189 | 0.046 | -4.107 | <0.001 |
| *Astreopora* | Δ Imperieuse | -0.059 | 0.043 | -1.387 | 0.166 |
| *Astreopora* | Δ Mermaid | -0.036 | 0.042 | -0.849 | 0.396 |
| *Astreopora* | Δ 2019 | 0.059 | 0.027 | 2.175 | 0.03 |
| Encrusting corals | Slope_Clerke_2018 | 3.607 | 0.953 | 3.783 | <0.001 |
| Encrusting corals | log (slope) | 1.941 | 0.214 | 9.08 | <0.001 |
| Encrusting corals | depth | -0.995 | 0.11 | -9.081 | <0.001 |
| Encrusting corals | Δ Crest | -3.916 | 0.535 | -7.325 | <0.001 |
| Encrusting corals | Δ Bommie | -5.959 | 0.785 | -7.587 | <0.001 |
| Encrusting corals | Δ Lagoon floor | -4.446 | 0.46 | -9.675 | <0.001 |
| Encrusting corals | Δ Imperieuse | 2.425 | 0.426 | 5.696 | <0.001 |
| Encrusting corals | Δ Mermaid | 4.677 | 0.417 | 11.211 | <0.001 |
| Encrusting corals | Δ 2019 | -1.142 | 0.27 | -4.228 | <0.001 |
| Foliose corals | Slope_Clerke_2018 | 0.966 | 0.224 | 4.319 | <0.001 |
| Foliose corals | log (images) | -0.138 | 0.094 | -1.458 | 0.145 |
| Foliose corals | depth | -0.131 | 0.029 | -4.592 | <0.001 |
| Foliose corals | Δ Crest | -0.429 | 0.142 | -3.026 | 0.003 |
| Foliose corals | Δ Bommie | -0.798 | 0.207 | -3.866 | <0.001 |
| Foliose corals | Δ Lagoon floor | -0.189 | 0.122 | -1.545 | 0.123 |
| Foliose corals | Δ Imperieuse | 0.177 | 0.105 | 1.69 | 0.091 |
| Foliose corals | Δ Mermaid | 0.247 | 0.104 | 2.369 | 0.018 |
| Foliose corals | Δ 2019 | -0.059 | 0.104 | -0.571 | 0.568 |
| *Isopora* | Slope_Clerke_2018 | -1.667 | 0.448 | -3.724 | <0.001 |
| *Isopora* | log (images) | -1.079 | 0.189 | -5.709 | <0.001 |
| *Isopora* | depth | 0.232 | 0.057 | 4.041 | <0.001 |
| *Isopora* | Δ Crest | 2.47 | 0.284 | 8.709 | <0.001 |
| *Isopora* | Δ Bommie | 1.784 | 0.413 | 4.315 | <0.001 |
| *Isopora* | Δ Lagoon floor | 0.491 | 0.245 | 2.004 | 0.045 |
| *Isopora* | Δ Imperieuse | -0.625 | 0.209 | -2.984 | 0.003 |
| *Isopora* | Δ Mermaid | -1.281 | 0.208 | -6.148 | <0.001 |
| *Isopora* | Δ 2019 | 0.789 | 0.207 | 3.807 | <0.001 |
| Merulinidae | Slope_Clerke_2018 | -2.73 | 0.505 | -5.403 | <0.001 |
| Merulinidae | log (slope) | 0.946 | 0.115 | 8.253 | <0.001 |
| Merulinidae | log (images) | -0.424 | 0.192 | -2.204 | 0.028 |
| Merulinidae | depth | 0.195 | 0.057 | 3.397 | <0.001 |
| Merulinidae | Δ Crest | 1.616 | 0.282 | 5.732 | <0.001 |
| Merulinidae | Δ Bommie | -0.115 | 0.411 | -0.28 | 0.78 |
| Merulinidae | Δ Lagoon floor | -1.239 | 0.242 | -5.112 | <0.001 |
| Merulinidae | Δ Imperieuse | 0.256 | 0.227 | 1.126 | 0.261 |
| Merulinidae | Δ Mermaid | -0.34 | 0.222 | -1.533 | 0.126 |
| Merulinidae | Δ 2019 | 0.716 | 0.208 | 3.449 | <0.001 |
| *Millepora* | Slope_Clerke_2018 | 0.05 | 0.384 | 0.13 | 0.897 |
| *Millepora* | log (images) | -0.454 | 0.209 | -2.176 | 0.03 |
| *Millepora* | log (slope) | 0.267 | 0.123 | 2.164 | 0.031 |
| *Millepora* | Δ Crest | -0.534 | 0.209 | -2.552 | 0.011 |
| *Millepora* | Δ Bommie | -0.887 | 0.257 | -3.447 | <0.001 |
| *Millepora* | Δ Lagoon floor | -1.129 | 0.254 | -4.441 | <0.001 |
| *Millepora* | Δ Imperieuse | 0.744 | 0.246 | 3.027 | 0.003 |
| *Millepora* | Δ Mermaid | -0.063 | 0.212 | -0.298 | 0.765 |
| *Millepora* | Δ 2019 | 0.476 | 0.226 | 2.107 | 0.035 |
| Pocilloporidae | Slope_Clerke_2018 | -0.739 | 0.289 | -2.559 | 0.011 |
| Pocilloporidae | log (images) | -1.004 | 0.157 | -6.4 | <0.001 |
| Pocilloporidae | log (slope) | 0.613 | 0.093 | 6.599 | <0.001 |
| Pocilloporidae | Δ Crest | -0.552 | 0.157 | -3.505 | <0.001 |
| Pocilloporidae | Δ Bommie | -0.146 | 0.193 | -0.754 | 0.451 |
| Pocilloporidae | Δ Lagoon floor | -0.971 | 0.191 | -5.077 | <0.001 |
| Pocilloporidae | Δ Imperieuse | -0.196 | 0.185 | -1.06 | 0.289 |
| Pocilloporidae | Δ Mermaid | -0.931 | 0.159 | -5.85 | <0.001 |
| Pocilloporidae | Δ 2019 | 0.733 | 0.17 | 4.316 | <0.001 |
| *Porites* | Slope_Clerke_2018 | -1.726 | 0.244 | -7.068 | <0.001 |
| *Porites* | log (slope) | 0.484 | 0.083 | 5.845 | <0.001 |
| *Porites* | Δ Crest | -0.215 | 0.135 | -1.592 | 0.112 |
| *Porites* | Δ Bommie | 0.721 | 0.168 | 4.296 | <0.001 |
| *Porites* | Δ Lagoon floor | 0.294 | 0.172 | 1.714 | 0.087 |
| *Porites* | Δ Imperieuse | 1.879 | 0.164 | 11.438 | <0.001 |
| *Porites* | Δ Mermaid | 0.116 | 0.139 | 0.837 | 0.403 |
| *Porites* | Δ 2019 | 0.248 | 0.106 | 2.345 | 0.019 |
| Uncommon | Slope_Clerke_2018 | -0.149 | 0.211 | -0.705 | 0.481 |
| Uncommon | log (slope) | 0.269 | 0.048 | 5.621 | <0.001 |
| Uncommon | log (images) | 0.306 | 0.08 | 3.81 | <0.001 |
| Uncommon | depth | -0.057 | 0.024 | -2.396 | 0.017 |
| Uncommon | Δ Crest | -0.197 | 0.118 | -1.676 | 0.094 |
| Uncommon | Δ Bommie | -0.333 | 0.171 | -1.942 | 0.053 |
| Uncommon | Δ Lagoon floor | 0.137 | 0.101 | 1.356 | 0.176 |
| Uncommon | Δ Imperieuse | 1.052 | 0.095 | 11.09 | <0.001 |
| Uncommon | Δ Mermaid | -0.003 | 0.093 | -0.035 | 0.972 |
| Uncommon | Δ 2019 | -0.545 | 0.087 | -6.281 | <0.001 |
